# Stepwise Interactive Situated Training Program for Young Nurses’ Safety Behavior and Interrupted Coping Behavior

**DOI:** 10.3390/healthcare10071157

**Published:** 2022-06-21

**Authors:** Jin Yan, Lijun Li, Jie Li, Sha Wang, Xiaoqi Wu, Panpan Xiao, Zhuqing Zhong, Siqing Ding, Jianfei Xie, Andy S. K. Cheng

**Affiliations:** 1 Nursing Department, The Third Xiangya Hospital of Central South University, Changsha 410000, China; yanjin0163@163.com (J.Y.); wangsha444562@163.com (S.W.); zhuqingzhong@csu.edu.cn (Z.Z.); sweettown@126.com (S.D.); 2 Xiangya Nursing School, Central South University, Changsha 410000, China; iamleelj@163.com (L.L.); 13297993158@163.com (J.L.); wxq12300828@163.com (X.W.); panpxiao@163.com (P.X.); 3 Department of Rehabilitation Sciences, The Hong Kong Polytechnic University, Hong Kong 999077, China; andy.cheng@polyu.edu.hk

**Keywords:** stepwise interactive situated training, young nurses, safety behavior, interrupted coping behavior

## Abstract

Young nurses’ safety behavior and interrupted coping behavior affect patient safety. A stepped, interactive and situated training program should be evaluated to assist young nurses in improving themselves. This study aimed to evaluate the effect of the stepwise interactive situated training program on safety behavior and practice ability with respect to nursing interruptions for young nurses and its influencing factors. This was a quasi-experimental, one-group, self-control and pretest–post-test design study. Six hundred young nurses in two provinces were included. The participants underwent a stepwise interactive situated training program from March to August 2019. The program was delivered by designated head nurses and consisted of five themes: mobilization, theoretical training, operational training, specialized training and self-improvement. Five hundred and sixty-two young nurses completed this study. The safety behavior and the practice of nursing interruption were significantly higher after intervention than before. Professional titles, age and occupational time were the influence factors. The stepwise interactive situated training program was effective at improving young nurses’ safety behavior and interrupted coping behavior. Nurses with higher professional titles performed better with regard to the safety behavior and the practice of nursing interruption.

## 1. Introduction

Both unsafe behavior and nursing interruptions affect patient safety and may result in adverse events [1,2,3,4], which occur each year due to unsafe nursing in hospitals in low- and middle-income countries, contributing to 2.6 million deaths annually. The issue of patient safety has attracted great attention from the World Health Organization and has become one of the most important issues in the field of hospital management [5].

Nurses’ safety behavior is an indispensable part of improving hospitals’ safety management, ensuring patient safety and preventing adverse events [6]. Unsafe nursing in the global health system is pervasive and seems to cause severe morbidity and mortality worldwide [7,8]. A total of 3% to 16% of hospitalized patients are harmed by healthcare in developed countries. The hazards of unsafe behavior include infections, incorrect medications or procedures, missed or delayed diagnoses and falls that were preventable in most situations [8,9,10].

Nursing interruption refers to unexpected events caused by the suspension of primary nursing tasks [11]. Interruptions are prevalent in the nursing work environment [12]. Brixey et al. reported that clinical nurses were interrupted more than six times per hour [13]. Approximately 89% of nursing interruptions could have negative consequences [14], such as leading to medication administration errors [15,16] and delaying working procedures [4,17].

Young nurses are those who have less than 10 years of service or who are younger than 30 years old. As the world’s nursing workforce ages, the health needs of its population grow. Recruiting and retaining young nurses is essential to replace nurses who will retire in the next 20 years [18]. Flinkman et al. reported that 26% of young nurses often thought about giving up their nursing careers in the past year [19], which was associated with stress, emotional exhaustion, poor opportunities for development, lack of advanced professional academic education, higher quantitative work requirements, etc. [20,21]. Previous studies have demonstrated that adverse events for second-victim nurses presented significant psychological distress consisting of widespread serious pessimistic emotions, thus indicating the magnitude of their feelings of culpability, and impacting their clinical performance. These experiences impugned their professional identity and, in some cases, caused continued psychological trauma [22,23]. The distress caused by adverse events was significantly correlated with turnover intentions [24].

Interactive situational training programs can reflect, anticipate, or amplify real-world situations with guided experiences in a fully interactive manner [25]. At present, interactive situational training for nurses can be based on the web or in the real environment. This approach has been widely applied in various nursing training programs, such as those focused on ethics, teamwork, clinical competence, confidence and nursing operations [26,27,28]. This kind of intervention strategy applies a theory-based educational approach to training, including demonstration, information processing, practice-based rehearsal and knowledge building. Stepwise interactive situated training programs can help young nurses gradually and effectively address actual clinical situations in today’s environment of different practices and improve safety quality.

Young nurses are the main workforce of the nursing team, accounting for more than 40% of nurses in Southeast Asia [29]. The systematic development and implementation of programs to enhance instructional behavior by nursing managers are essential to improving patients’ safety and nursing [30]. Few studies have applied stepped training for young nurses and concentrated on safety behavior and interrupted coping strategies, which are essential elements for patient safety.

We constructed the intervention based on the Self-Efficacy Theory and the Revised Bloom’s Taxonomy of Education Objectives. The Self-Efficacy Theory was proposed by an American psychologist [31]. It was believed that effective approaches to change self-efficacy include performance accomplishments, vicarious experience, verbal persuasion and emotional arousal. Intervention measures at various stages of the step interactive situational training were developed based on the above four aspects. The Revised Bloom Taxonomy of Educational Objectives is used for the specific implementation of the two phases of operative training and mentor-based interactive face-to-face instruction, including objectives, teaching methods and cognitive process-centered assessment to improve young nurses’ ability to recognize, identify and solve clinical nursing problems [32,33,34,35]. Therefore, we developed a stepwise interactive situated training (SIST) program.

The purpose of this study was to (1) evaluate the effect of the step interactive situated training program on the safety behavior and interrupted coping behavior of young nurses through before-and-after control and (2) detect the influencing factors of young nurses’ safety behavior and interrupted coping behavior.

## 2. Methods

### 2.1. Design

This was a quasi-experimental, one-group, self-control and pretest–post-test design study.

### 2.2. Sample and Setting

According to the availability of advanced medical equipment, the number of hospital beds, the physical area of the medical school, and public hospitals are divided into three levels (1st, 2nd, 3rd). The 1st level hospitals are primary healthcare units located in the countryside or near small communities. The 2nd level hospitals are mainly located in small and medium-sized cities with between 100 and 800 inpatient beds, and provide healthcare services for the majority of patients in China. The 3rd level hospitals are inter-regional, provincial, municipal and national hospitals with more than 500 inpatient beds each. It is a medical prevention technology center integrating medicine, teaching and research [36]. We enrolled 600 young nurses in two comprehensive 3rd level hospitals. The 3rd level hospitals are the standard representatives of Chinese hospitals. Their rules and regulations are more perfect and reasonable. Therefore, this study mainly concentrated on two 3rd level hospitals, which were selected by convenience sampling. Young nurses were recruited through management meeting releases and gave informed consent.

**Figure 1:**
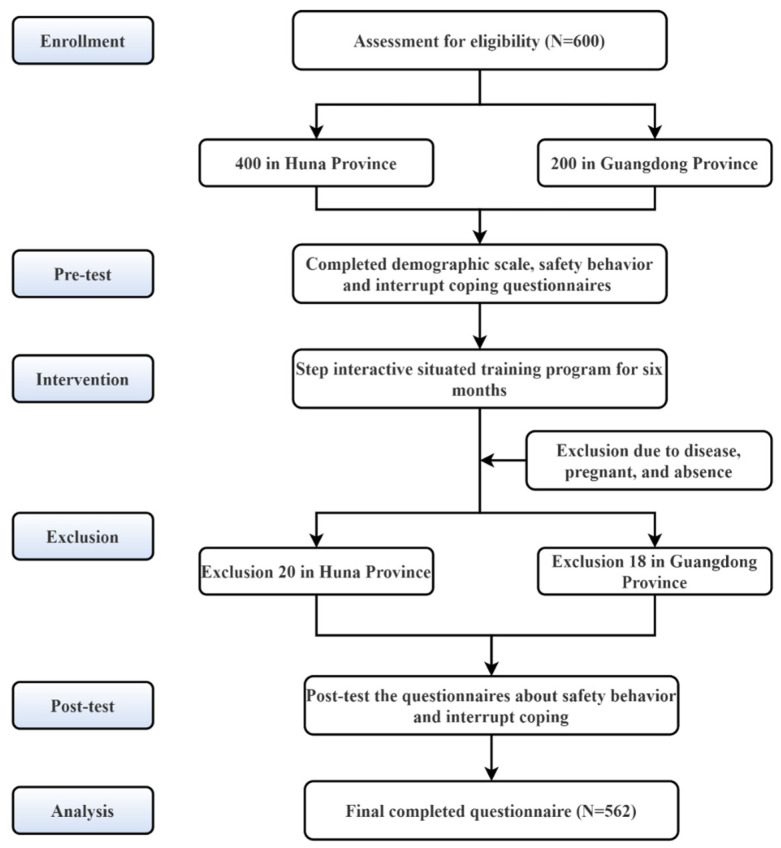
The flowchart of the study.

The intervention period was from March to August 2019. The inclusion criteria were as follows: (1) nurses with less than 10 years of service or under 30 years of age; (2) occupational age of more than 1 year; (3) full attendance or on duty in the next year; and (4) registered nurse. This study was performed among direct care nurses from clinical units, excluding drug distribution centers and disinfection supply centers (Figure 1). All participants were numbered before collecting data. The number was applied for identification before and after the intervention while the anonymity of participants was guaranteed.

### 2.3. Intervention Process

**Figure 2:**
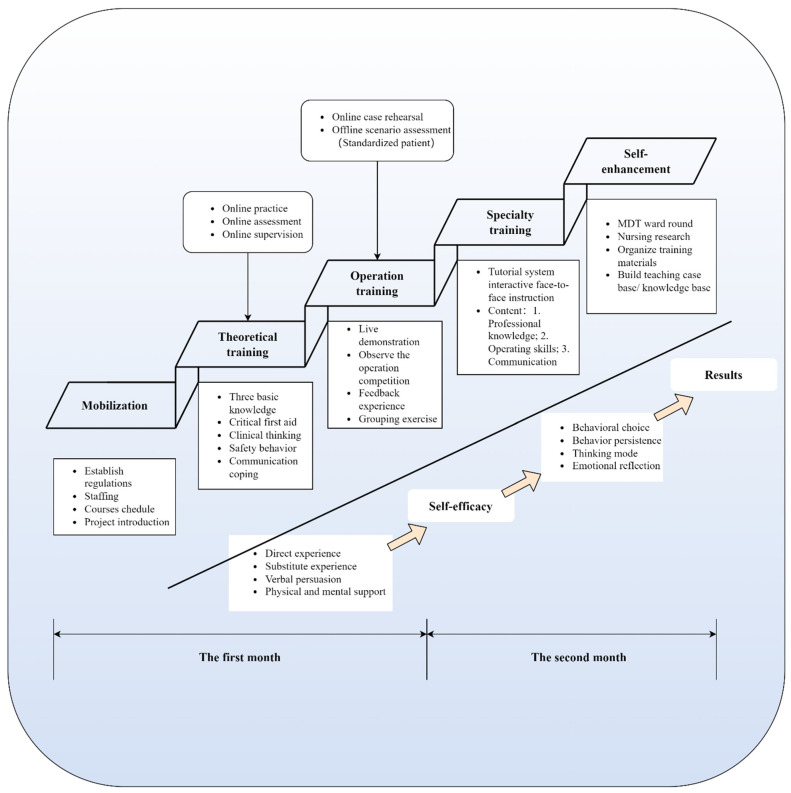
The step interactive situated training program. Abbreviations: MDT, multidisciplinary team.

The SIST includes 5 training topics: mobilization, theoretical training, operational training, specialized training and self-improvement (Figure 2). Eight educators from each of the two hospitals participated: two professors, four associate professors and two nursing teaching cadre nurses. Educators worked together to design the curriculum and standardize the process of educational delivery. Young nurses are trained together through the same curriculum, using the same teaching staff. Homogenization of course information and quality are guaranteed. This study set up a centralized training for every 200 young nurses, and at the end of the training a feedback discussion was organized for groups of 10–12 people. The young nurses completed the demographic characteristics scale, the Safety Behavior Scale (SBS) and the practice subscale of the Knowledge, Attitude and Practice Questionnaire of Nursing Interruptions (KAP). The mobilization meeting introduced the regulations, staffing, course schedule and project. This step familiarized the participants with the researchers and allowed participants to have a preliminary understanding of the intervention project. In the first month, researchers conducted online theoretical training and case previews for participants and offline situation assessment. The theoretical training included three basic areas of knowledge: critical first aid, clinical thinking, safety behavior and communication coping. Operation training was carried out through the methods of a live demonstration, observation of operation competition, feedback experience and group exercises. Specialty training followed in the second month. Interactive face-to-face instruction via a tutorial system supported this phase to provide content on professional knowledge, operating skills and communication. At the end of the study, the topic of self-improvement included multidisciplinary ward rounds and nursing research. We also organized the training materials and built a teaching case base or knowledge base to optimize this training program.

Additionally, all participants joined in this study during non-working hours. Operational training was divided into three groups, including clinical cases in medicine, surgery and pediatrics, and each category contained two types of basic nursing operations. The designated head nurses were responsible for delivering the program in the Hospital Nursing Teaching and Research Classroom. To understand the impact of the step interactive situated training program on clinical safety behavior and interrupted coping behavior in youth nurses, we performed measurements again at the end of the intervention.

### 2.4. Instruments

#### 2.4.1. Demographic Characteristics

A five-item demographic questionnaire was applied to collect information on gender, age, highest education level, professional title and working year.

#### 2.4.2. Safety Behavior Scale (SBS)

Based on the original safety behavior questionnaire [37], the Chinese version was revised by Rong et al. [38]. Then the questionnaire was updated with 9 items [39]. The SBS can determine nurses’ behaviors in their work that prevent patient harm or improve patient safety, scored on a 5-point Likert scale (never = 1, always= 5). A higher score indicated that the nurses performed better in terms of patient safety. The Cronbach’s α coefficient of the 9-item SBS in nurses was 0.805 and is widely applied [39].

#### 2.4.3. Knowledge, Attitude and Practice Questionnaire of Nursing Interruptions (KAP)

Xie et al. designed the (KAP), which contains three dimensions: knowledge, attitude and practice [40]. In this study, we only used the 7-item practice dimension of nursing interruptions (P-KAP) to measure interrupted coping behavior. It included items on nursing interruption identification, training, treatment and management, with a score range of 1–4 for each item. A high score on the practice subscale represents positive practices. The Cronbach’s α, retest reliability and Spearman–Brown split-half coefficient were 0.953, 0.850–0.890 and 0.986, respectively. The reliability and validity were satisfactory in Chinese nurses [40].

### 2.5. Data Collection

This study collected data at two times. Researchers spent 3 days collecting all data with an electronic questionnaire within three days before and after intervention.

### 2.6. Ethics Approval and Consent to Participate

This study followed throughout the ethical principles of the Declaration of Helsinki, and was approved by the Ethics Committee of The Third Xiangya Hospital of Central South University (NO. 2017-S559). All young nurses signed an informed consent when agreeing to participate in the study.

### 2.7. Data Collection and Analysis

Data were collected in two stages: before and after the intervention. The researchers collected data on-site after training. We used SPSS 26.0 (IBM Corp., Armonk, NY, USA) for data import and analysis. Frequencies, percentages and means ± standard deviations were applied to describe the demographic information and the scales’ scores. The samples were tested by the Shapiro–Wilk normal test. The data presented a non-normal distribution. Spearman’s correlation analysis was used to analyze the relationship between the continuous independent variable and the results. We used the Wilcoxon signed-rank test to analyze the difference between the pretest and post-test values for the main variables. A *p*-value of less than 0.05 was considered significant.

## 3. Results

### 3.1. Demographic Characteristics and Their Correlation with Safety Behavior and Interrupted Coping Behavior

A total of 600 participants were recruited in the first instance, and 562 (93.67%) young nurses participated in the entire study. The majority of them were female (92.3%), had bachelor’s degrees (85.4%) and were senior nurses (a kind of professional title) (64.1%). The mean age and occupational age were 27.22 ± 2.67 and 5.37 ± 2.99, respectively. We found that gender may affect the safety behavior of young nurses in the pretest (*p* = 0.001). Females (3.54 ± 0.52) had a higher score than males (3.30 ± 0.47). However, the male participants were few. Meanwhile, both before and after the intervention, there was a significant difference between professional title and safety behavior or the practice dimension of the KAP (*p* < 0.05). Young nurses with higher professional titles performed better (Table 1).

### 3.2. The Correlation between Age or Occupational Age and Safety Behavior or Interrupted Coping Behavior

**Figure 3:**
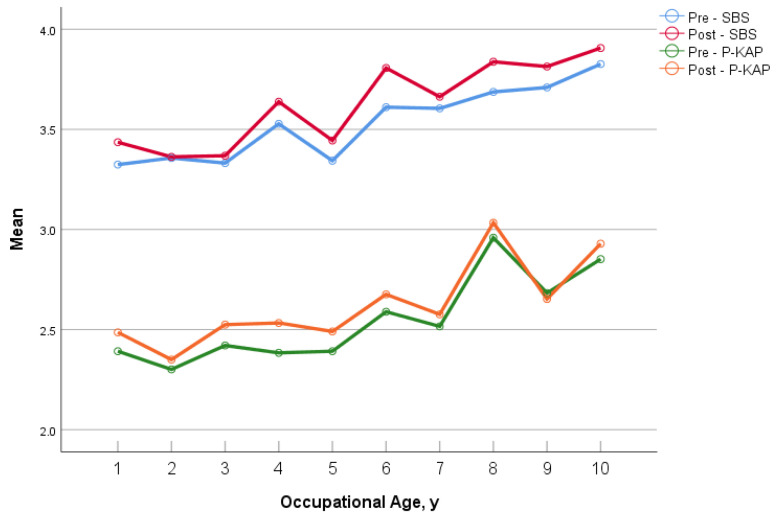

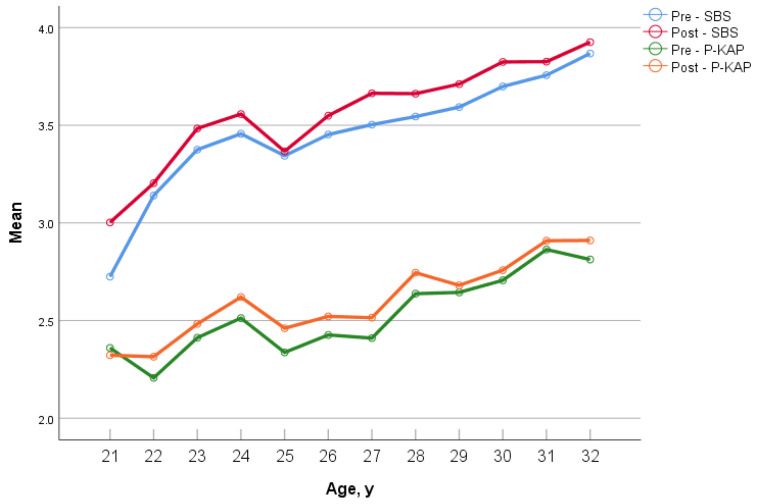
The correlation between age, occupation and SBS or P-KAP. Abbreviations: SBS, Safety Behavior Scale; P-KAP, Practice dimension of Knowledge, Attitude and Practice Questionnaire of Nursing Interruptions.

As shown in Table 2, we found that age and occupational age were positively correlated with young nurses’ safety behavior and interrupted coping behavior (*p* < 0.001). The correlations increased after the intervention. The linear trends between the age or occupational age and the SBS or P-KAP of the participants are shown in Figure 3. The overall scores after the intervention were higher than those before the intervention.

Additionally, the young nurses’ professional title was highly correlated with age (r = 0.74, *p* = 0.000) and occupational age (r = 0.74, *p* < 0.001). The elderly participants had worked for a longer period (r = 0.89, *p* < 0.001) (see Table 3).

### 3.3. The Difference between the Pretest and Post-Test Scores on the SBS and P-KAP

As shown in Table 4, the scores of young nurses in safety behaviors (*p* < 0.001) and practice dimensions of nursing interruptions (*p* < 0.001) were significantly different between the pretest and posttest.

## 4. Discussion

Both before and after the intervention, the scores on the SBS and the P-KAP increased with participants’ promotions in the professional title, increased age or occupational age. Meanwhile, we also found that the older the nurses’ ages and the longer their length of service, the higher their professional titles were. Nurses with higher professional titles have a longer length of nursing experience, a stronger awareness of adverse event risk perception and more experience in addressing emergencies, forming a positive feedback system to promote their safety behavior [41,42]. They also might contribute to reducing the incidence of major complications to improve medical outcomes [43]. Workers with a higher occupational age have established more complex conventions than newcomers have and may be more effective at handling interruptions and multitasking demands [44].

Young nurses’ safety behavior was similar to that observed in a previous study [45], which showed that nurses with higher professional titles engaged in better safety compliance behavior. Although few studies have concentrated on young nurses’ professional titles and safety behavior, previous studies have reported that professional title is a significant predictor of corresponding knowledge, leadership and nursing competence [42,46,47,48]. With regard to the interrupted coping behavior, professional titles were associated with nursing interruptions, including practice competence [40,49]. As nursing age increased, the more work experience that nurses accumulated, and the more likely they were to be promoted to higher professional titles. Their safety awareness was higher, and it was easier to identify the hidden safety hazards of patients in the medical system and address these problems in a timely and efficient manner. Nursing managers should mobilize the enthusiasm and initiative of more experienced nurses and encourage them to offer suggestions for patient safety management. At the same time, the education and training of young nurses with low professional titles should be strengthened to practically improve the safety behavior of clinical nurses.

The SIST program has significantly improved young nurses’ safety behavior and interrupted coping, which directly affects patient safety [4,50]. This training process allowed participants to accumulate direct and alternative experiences. It is critical to improve young nurses’ behavioral choices and persistence at work and cultivate their thinking patterns and emotional reflection [31]. The classification of educational objectives was utilized for the specific implementation of operational training and the interactive face-to-face guidance of the tutorial system, including the objectives, teaching methods and the cognitive-process-centered assessment [33,34]. The above processes contribute to young nurses’ behavioral choices, behavior persistence, thinking mode construction and emotional responses. Interactive situations contribute to integrated thinking, knowledge transfer and an improved ability of a theory to be put into practice [51]. Furthermore, situation-based teaching provides an alternative to learning environments without compromising patient safety [22]. This study showed that the two-month SIST program for young nurses improved their clinical safety behavior and practice ability with respect to nursing interruptions. In the first month of this training program, we concentrated on the reinforcement of participants’ theoretical knowledge and nursing operational knowledge. This period was the primary step of the program, which aimed to improve the basic theory and operation of young nurses through online and offline modes. In the second month, the preceptors’ interaction system was an essential element for young nurses to improve themselves. Young nurses faced difficulties in career development [18], and the SIST program is an effective learning tool for helping young nurses to acquire safety knowledge, independently solve clinical problems and enhance self-worth.

This study provided a stepped and practical program for nursing management or educators to support the enhancement of young nurses’ safety behavior and interrupted coping behavior. The SIST program provides the following highlighted and personalized approaches to assist young nurses in their career development and prevent the incidence of adverse events. First, the stepwise training mode gradually helps young nurses improve their comprehensive ability. In addition, the program combines online and offline approaches. Second, young nurses can interact with preceptors face-to-face. The preceptorship can provide help, clinical support and feedback to nurses [52]. Finally, the situated training mode promotes mutual learning between participants and preceptors. Through situational learning, nurses can have opportunities for practice, develop skills, increase knowledge and increase self-confidence in a clinical setting without harming the patient. They can also play different roles, strengthen their thinking patterns and improve their ability to choose behaviors in different situations [53,54].

### Limitations

The participants’ safety behavior and interrupted coping behavior were significantly improved after this program. However, there were a few limitations in this study. Firstly, this study was a pre-and-post quasi-experimental study with no control group for comparison during the same period. Secondly, this study was confined to a specific region and the short follow-up period (2 months). A large-scale study with a longer follow-up may be necessary to enrich the current results.

## 5. Conclusions

After the two-month SIST program was completed, the head nurses and young nurses were satisfied with the training content, time, form, feedback and effect. The SIST program was effective at improving young nurses’ safety behavior and interrupted coping behavior. Young nurses with higher professional titles performed better on the SBS and the practice subscale of the KAP.

## Figures and Tables

**Table 1:** The demographic characteristics of participants and the scores of measurements (N = 562).

Characteristics	N (%)/Mean ± SD	SBS		*p* (Difference in Pre-/Post-)	Practice Subscale of KAP		*p* (Difference in Pre-/Post-)
		Pro-Test	Post-Test		Pro-Test	Post-Test	
Gender				<0.001/0.455			0.274/0.130
Male	43 (7.7)	3.30 ± 0.47	3.68 ± 0.66		2.40 ± 0.39	2.58 ± 0.46	
Female	519 (92.3)	3.54 ± 0.52	3.73 ± 0.63		5.56 ± 0.57	2.72 ± 0.55	
Highest Education				0.453/0.611			0.226/0.907
Junior college or below	55 (9.8)	3.58 ± 0.63	3.77 ± 0.70		2.63 ± 0.59	2.72 ± 0.56	
Bachelor	480 (85.4)	3.51 ± 0.51	3.72 ± 0.62		2.54 ± 0.55	2.71 ± 0.53	
Master or above	27 (4.8)	3.64 ± 0.40	3.83 ± 0.59		2.50 ± 0.62	2.80 ± 0.68	
Professional title				<0.001/<0.001			<0.001/0.023
Nurse	74 (13.2)	3.23 ± 0.64	3.41 ± 0.67		2.41 ± 0.50	2.62 ± 0.54	
Senior nurse	360 (64.1)	3.51 ± 0.48	3.73 ± 0.62		2.50 ± 0.50	2.69 ± 0.51	
Supervisor nurse	128 (22.8)	3.74 ± 0.46	3.91 ± 0.55		2.75 ± 0.68	2.83 ± 0.63	
Age, y	27.22 ± 2.668	/	/	/	/	/	/
Occupational age, y	5.37 ± 2.990	/	/	/	/	/	/

Abbreviations: SBS, Safety Behavior Scale; KAP, Knowledge, Attitude and Practice Questionnaire of Nursing Interruptions.

**Table 2:** The correlation between age or occupational age and safety behavior or interrupt coping behavior (N = 562).

Characteristics	SBS/r		*p*	P-KAP/r		*p*
	Pro-Test	Post-Test		Pro-Test	Post-Test	
Age, y	0.25	0.27	<0.001	0.22	0.25	<0.001
Occupational age, y	0.28	0.30	<0.001	0.24	0.25	<0.001

Abbreviations: SBS, Safety Behavior Scale; P-KAP, Practice dimension of Knowledge, Attitude and Practice Questionnaire of Nursing Interruptions.

**Table 3:** The correlation between professional title, age and occupational time (N = 562).

	Professional Title	Age, y	Occupational Age, y
Professional title	--	0.74 ^a^	0.72 ^a^
Age, y		--	0.89 ^a^
Occupational age, y			--

^a^*p* < 0.05.

**Table 4:** Comparison of SBS and P-KAP scores between pre- and post-intervention (N = 562).

Scales	Mean ± SD		Z	*p*
	Pre-Test	Post-Test		
SBS	3.53 ± 0.52	3.72 ± 0.63	−11.87	<0.001
Item 1—I will try to make patients feel safest during work.	3.64 ± 0.73	3.79 ± 0.72	−6.85	<0.001
Item 2—I will prioritize patient safety when making difficult judgments.	3.72 ± 0.58	3.88 ± 0.66	−7.18	<0.001
Item 3—I will try to adjust the mental and physical strength to the most suitable state at work.	3.83 ± 0.61	3.96 ± 0.67	−7.25	<0.001
Item 4—I will read the standard workflow established by the unit before taking care of patients.	3.05 ± 0.51	3.26 ± 0.71	−8.23	<0.001
Item 5—I will carefully follow the unit’s operating instructions on the standard safety workflow.	3.04 ± 0.48	3.23 ± 0.69	−7.22	<0.001
Item 6—I will cause no accidents.	3.66 ± 0.74	3.84 ± 0.77	−7.63	<0.001
Item 7—I will ask my boss or supervisor when in doubt.	3.81 ± 0.65	3.95 ± 0.68	−7.52	<0.001
Item 8—I will clean my hands before touching the patients.	3.45 ± 0.70	3.81 ± 0.76	−11.67	<0.001
Item 9—I always take the initiative to confirm the patient’s identity when it involves any treatment and patient care.	3.51 ± 0.71	3.84 ± 0.76	−10.66	<0.001
Practice subscale of KAP	2.54 ± 0.56	2.71 ± 0.54	−11.56	<0.001

Abbreviations: SBS, Safety Behavior Scale; P-KAP, Practice dimension of Knowledge, Attitude and Practice Questionnaire of Nursing Interruptions.

## Data Availability

The data presented in this study are available on request from the corresponding author. The data are not publicly available due to ethical restrictions.
